# Patients with Clinically Suspected Gallstone Disease: A More Selective Ultrasound May Improve Treatment Related Outcomes

**DOI:** 10.3390/jcm12124162

**Published:** 2023-06-20

**Authors:** Floris M. Thunnissen, Daan J. Comes, Remy W. F. Geenen, Deniece Riviere, Carmen S. S. Latenstein, Marten A. Lantinga, Henk J. Schers, Cornelis J. H. M. van Laarhoven, Joost P. H. Drenth, Femke Atsma, Philip R. de Reuver

**Affiliations:** 1 Department of Surgery, Radboud Institute for Health Sciences, Radboud University Medical Center, 6525 GA Nijmegen, The Netherlands; 2 Department of Radiology, Northwest Clinics, 1815 JD Alkmaar, The Netherlands; 3 Department of Radiology, Jeroen Bosch Hospital, 5223 GZ ‘s-Hertogenbosch, The Netherlands; 4 Department of Surgery, Amsterdam UMC, Location VUMC, 1081 HV Amsterdam, The Netherlands; 5 Department of Gastroenterology and Hepatology, Amsterdam UMC, Location AMC, 1105 AZ Amsterdam, The Netherlands; 6 Department of General Practice, Radboud Institute for Health Sciences, Radboud University Medical Center, 6525 GA Nijmegen, The Netherlands; 7 Department of Gastroenterology and Hepatology, Radboud Institute for Health Sciences, Radboud University Medical Center, 6525 GA Nijmegen, The Netherlands; 8 Radboud Institute for Health Sciences, Scientific Center for Quality of Healthcare, Radboud University Medical Center, 6525 GA Nijmegen, The Netherlands

**Keywords:** cholecystolithiasis, gallstone disease, upper abdominal pain, ultrasound, general practitioner

## Abstract

This study aimed to quantify the confirmation of gallstones on ultrasound (US) in patients with suspicion of gallstone disease. To aid general practitioners (GPs) in diagnostic workup, a model to predict gallstones was developed. A prospective cohort study was conducted in two Dutch general hospitals. Patients (≥18 years) were eligible for inclusion when referred by GPs for US with suspicion of gallstones. The primary outcome was the confirmation of gallstones on US. A multivariable regression model was developed to predict the presence of gallstones. In total, 177 patients were referred with a clinical suspicion of gallstones. Gallstones were found in 64 of 177 patients (36.2%). Patients with gallstones reported higher pain scores (VAS 8.0 vs. 6.0, *p* < 0.001), less frequent pain (21.9% vs. 54.9%, *p* < 0.001), and more often met criteria for biliary colic (62.5% vs. 44.2%, *p* = 0.023). Predictors for the presence of gallstones were a higher pain score, frequency of pain less than weekly, biliary colic, and an absence of heartburn. The model showed good discrimination between patients with and without gallstones (C-statistic 0.73, range: 0.68–0.76). Clinical diagnosis of symptomatic gallstone disease is challenging. The model developed in this study may aid in the selection of patients for referral and improve treatment related outcomes.

## 1. Introduction

Key messages:

- Gallstones were not found on US in two-thirds of patients referred for a suspicion of gallstone disease.
- Variables associated with gallstones were biliary colic, more severe pain, frequency of pain less than weekly, and an absence of heartburn.
- A prediction model may aid the GP in the selection of patients for US in case of suspicion of gallstones.

The occurrence of gallstone disease is a significant and rising health issue in Western society [1]. International guidelines advise laparoscopic cholecystectomy as the treatment for symptomatic and complicated cholecystolithiasis [2,3,4]. Laparoscopic cholecystectomy is the most commonly performed gastrointestinal surgical procedure, resulting in 80,000 surgeries in the United Kingdom and 24,000 in the Netherlands annually [5,6]. In the Netherlands, the surgery rate of patients with symptomatic gallstone disease is approximately 70% [7]. Symptomatic cholecystolithiasis is an ill-defined condition ranging from epigastric pain to steady, nonparoxysmal pain in the presence of gallstones on transabdominal ultrasound (US) [8]. Due to underlying functional gastrointestinal diseases (FGID) and lack of consensus on patient selection, up to 40 percent of patients report persisting upper abdominal pain (UAP) after cholecystectomy [9,10]. 

The SECURE trial has shown that fulfilment of the ROME III criteria for biliary colic (severe steady pain, lasting 30 min or more, located in the epigastrium and/or right upper quadrant) is a poor predictor of pain relief after cholecystectomy [9]. This recently performed multicentre RCT showed that a restrictive strategy, based on the selection for surgery on the ROME III criteria, results in 8% fewer cholecystectomies but more patients with persistent pain [9]. The outcome of the trial emphasized the need for better criteria to select patients that will benefit from surgery. A recent analysis of Dutch primary care registry data from 633 patients showed that more than half of the patients referred for treatment of gallstones return to their general practitioner for persistent abdominal symptoms irrespective of cholecystectomy [11]. The high number of returning patients illustrates the importance of better selecting patients for surgery early in their trajectory. Patients’ expectations of diagnostics and treatment influence decision making and play an essential role in patient reported outcomes [12,13]. 

To aid in the diagnostic workup and decision making in patients with UAP and clinically suspected gallstone disease, the present study aims to quantify the outcome of US. Secondly, a multivariable regression model was developed to predict the occurrence of gallstones. Patient selection based on easily available patient characteristics and symptoms would be of considerable value for clinical decision making and patient expectations.

## 2. Materials and Methods

### 2.1. Study Design

This prospective cohort study was conducted in patients with a clinical suspicion of gallstone disease who were referred for an US under direct responsibility of the GP at the Department of Radiology of two general hospitals in The Netherlands (Jeroen Bosch Hospital, ‘s-Hertogenbosch and Northwest clinics, Alkmaar). Patients were approached for participation between May 2018 and July 2020. Patients ≥18 years of age who were clinically suspected of gallstone disease by their GP were eligible for inclusion. Patients were not eligible for inclusion if the clinical suspicion of gallstone disease was not stated in the referral letter, they had a history of cholecystectomy, or were referred for US for another reason. Additionally, the exclusion criteria were a history of complicated cholecystolithiasis (i.e., choledocholithiasis, acute cholecystitis, biliary pancreatitis, or cholangitis), previous malignancy, pregnancy, active mental disorder or incompetence that influences reliability of questionnaire response, or insufficient knowledge of the Dutch language.

This study was approved by the medical ethics commission (METC: 2017-3306) and performed in accordance with the ethical standards of the updated Helsinki Declaration of 2013. We followed Strengthening the Reporting of Observational studies in Epidemiology (STROBE) guidelines [14]. 

### 2.2. Recruitment and Selection of Study Subjects

Patients were contacted by telephone before the appointment for a US to assess in- and exclusion criteria and to discuss consent for participation. All eligible patients who gave informed consent received a paper or digital baseline questionnaire. Follow-up questionnaires were sent after six months. A reminder at baseline and follow-up was sent to participants who did not respond.

### 2.3. Measurements

The questionnaire consisted of the Izbicki Pain Score (IPS) and questions on abdominal symptoms. The IPS consists of four questions regarding the frequency of pain, intensity of pain based on a Visual Analog Scale (VAS score: 0 = no pain and 10 = worst imaginable pain), and use of pain medication and disease-related inability to work [15]. The IPS score is expressed as continuous pain scale (IPS score: 0 = no pain and 100 = worst imaginable pain).

After 6 months, follow-up clinical data were obtained from the medical records on health care consumption in the secondary care level. Clinical data and health care consumption in primary care were not collected.

### 2.4. Outcomes

The primary outcome was the occurrence of gallstones or sludge on US in patients with a clinical suspicion of gallstone disease according to the GP. Clinical suspicion of gallstones was scored when a GP referred a patient with the question of possible gallstone disease; symptomatology was generally not, or very briefly, reported on the referral. US-proven gallstones included positive findings of gallstones or sludge in the gallbladder. The US report was retrieved from patients’ medical record from the hospital. Secondary endpoints were comparisons between patients with and without gallstones treatment-related outcomes at six months.

A prediction model was developed to assess predictive factors for the presence of gallstones and/or sludge on US in patients with a clinical suspicion of gallstone disease as stated by the GP. In order to ensure sufficient power, a minimum of 10 outcome events per selected predictor variable was applied as a rule of thumb (EPV) [16]. Seven variables collected at baseline were entered into the model: biliary colic according to ROME III-criteria, pain characteristics (pain radiation to the back, use of simple anaesthetics, frequency of pain, and VAS pain score), heartburn, and nausea. The VAS pain score was entered as squared function because of nonlinearity. Baseline predictor information was retrieved from the survey at baseline. Discrimination was quantified using the area under the curve (AUC). 

The number of cholecystectomies and pain-free patients at six months of follow-up were assessed by the survey. The definitions of pain or pain-free are in line with previous SECURE trials and SUCCESS trials: IPS score ≤ 10 and a VAS pain score ≤ 4 [9,15,17].

### 2.5. Statistical Analysis

Missing data at baseline were imputed using a multiple imputation strategy (30 sets) and predictive mean matching for continuous variables [18]. Patients with missing data in follow-up questionnaires after six months were excluded from the follow-up analyses, instead of using multiple imputation, because the outcomes at baseline were defined as the primary outcome. Responders and nonresponders were compared on baseline characteristics (e.g., sex, age, and presence of gallstones), along with patients who did and did not withdraw their consent.

The baseline characteristics (age and BMI) and information from the survey (pain scores and abdominal symptoms) were presented as mean with standard deviation when normally distributed and median with range or inter quartile range (IQR) when the data were not normally distributed. Dichotomous data were summarized by frequencies and proportions (pain characteristics, fulfilment of the ROME criteria, and abdominal symptoms). Differences between patients with and without gallstones were analysed using the chi-squared test, Fisher’s exact test, independent *t*-test, and Mann–Whitney U test, depending on the type of data. The normality of data distributions was based on the Shapiro–Wilk test. *p*-values of less than 0.05 indicated statistical significance.

A multivariable logistic regression analysis with backward selection was performed to assess predictors at baseline for the detection of gallstones in patients. US-confirmed gallstones yes/no served as the outcome in this model. The independent variables were selected based on significant differences in descriptive analyses and clinical relevance. The model’s performance was assessed based upon its discrimination ability to differentiate between patients with and without gallstones. Based on the recent findings regarding the ROME III criteria in the SECURE trial, the discrimination ability of the model with solely the ROME III criteria was assessed [8,9]. The threshold for variable selection was 0.15. Receiver operating characteristic curve (ROC) with area under the curve (AUC) analysis was used to evaluate the performance of the model to differentiate between patients with and without gallstones. Data analysis was performed using IBM SPSS, version 27 (SPSS Inc., Chicago, IL, USA).

## 3. Results

### 3.1. Patient’s Characteristics

**Figure 1:**
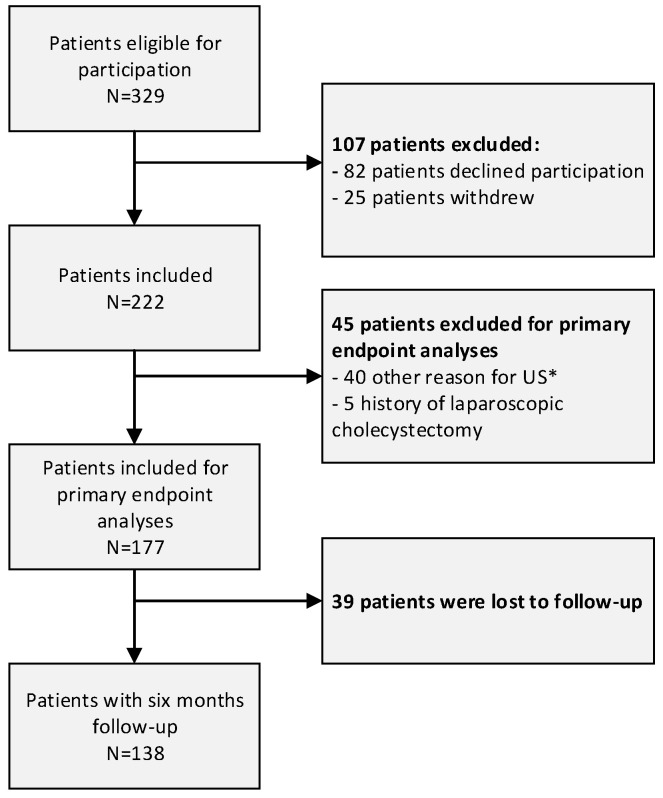
Flowchart of included patients with clinical suspicion of gallstone disease. Patients ≥ 18 years of age with clinical suspicion of gallstone disease who were referred by their GP for a direct-access US were eligible for primary endpoint analyses. * 40 patients were excluded because they were referred for US for: 15 patients with missing of a clinical suspicion of gallstones, 8 patients for suspected malignancy, 10 patients for abdominal wall hernia or benign process, 3 patients for follow-up of gallbladder polyp(s), 2 patients for left abdominal wall pain, 1 patient for lower abdominal pain and 1 patient for suspected urinary tract deficiency.

In total, 329 patients were assessed for eligibility. Forty-five patients (13.7%) were not eligible for inclusion due to the following: no stated clinical suspicion of gallstone disease in referral letter (N = 15), patients with a history of cholecystectomy (N = 5), or other reason for US (N = 25). Of the 284 included patients, 82 patients (28.9%) declined to participate and 25 patients (8.8%) withdrew informed consent. No significant differences were found in basic patient characteristics (i.e., sex and age) regarding included patients and patients who withdrew informed consent. This resulted in a final study sample of 177 patients (Figure 1). The characteristics of patients included for analyses are shown in Table 1.

### 3.2. Confirmation of Gallstones on Ultrasound in Patients with Clinical Suspicion of Gallstone Disease

In total, 177 patients were specifically referred for US because of a clinical suspicion of gallstones. In 64 of these 177 patients (36.2%), the presence of gallstones was confirmed using US. Sludge was found in 4.7% and a thickened gallbladder wall in 2.8% of patients with gallstones.

### 3.3. Comparison of Patients with and without Gallstones

In Table 1, baseline characteristics of patients with (N= 64) and without (N = 113) gallstones are compared. Patients with gallstones reported more severe pain (VAS score: median 8.0 (IQR 5.5–9.0) vs. 6.0 (IQR 4.0–7.5), *p* < 0.001), which occurred less frequently (weekly pain: 21.9% vs. 54.9%, *p* < 0.001), and for which they used pain medication more often (56.3% vs. 33.6%, *p* = 0.003). Patients with gallstones met the criteria for a biliary colic more often (62.5% vs. 44.2%, *p* = 0.023) compared to patients without gallstones on US. Patients without gallstones reported more abdominal bloating compared to patients with gallstones (22.1% vs. 7.8, *p* = 0.026). Age, sex, and BMI were comparable between the two groups.

### 3.4. Prediction Model

**Figure 2:**
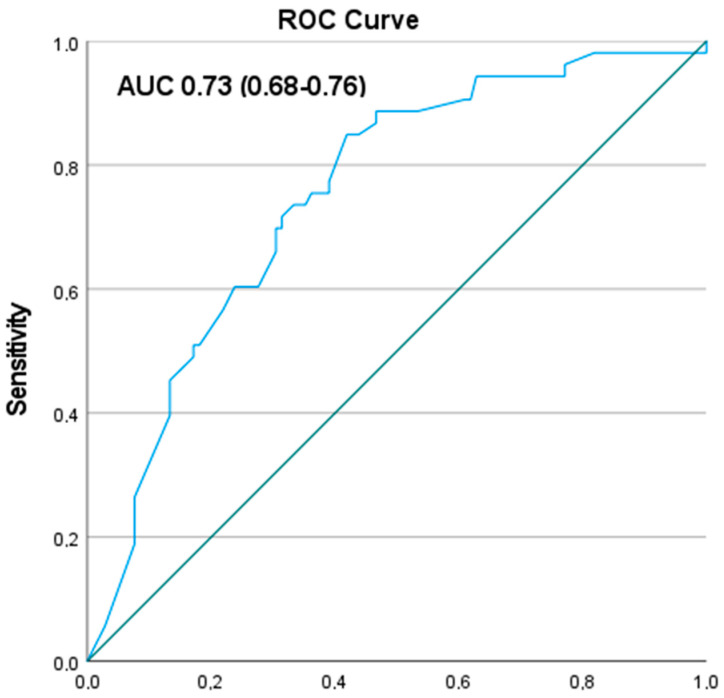
Calibration plot of the prediction model for the presence of gallstones in patients with upper abdominal pain. Calibration plot of the prediction model for presence of gallstones on abdominal ultrasound in patients with clinical suspicion of gallstone disease. The green line is the ideal line. The blue line is the area under the curve (AUC).

The prediction model for gallstones on US included the following variables: biliary colic in accordance with the ROME III criteria, absence of heartburn, frequency of pain, and VAS pain score. It showed good discriminative ability (AUC 0.73, range 0.68–0.76) (Figure 2). Regression coefficients and odds ratios of the model are shown in Table 2. The discriminative ability of the ROME III criteria for biliary colic alone was poor (AUC 0.59, range 0.57–0.61).

### 3.5. Outcomes at Six Months’ Follow-Up

Of all patients, 77.9% (138/177) responded to the follow-up questionnaire at 6 months. No significant differences were found between responders and nonresponders in terms of age, sex, or the presence of gallstones. A total of 39 of 138 patients (28.3%) reported persisted abdominal pain. During follow-up, 49 of 64 (76.5%) patients with gallstones were referred to the surgical outpatient clinic, of whom 36 (73.5%) underwent laparoscopic cholecystectomy. At six months follow-up, patients without gallstones reported more persisted pain compared to patients with gallstones (37.2% vs. 13.5%, *p* = 0.003).

## 4. Discussion

### 4.1. Main Findings

**Figure 3:**
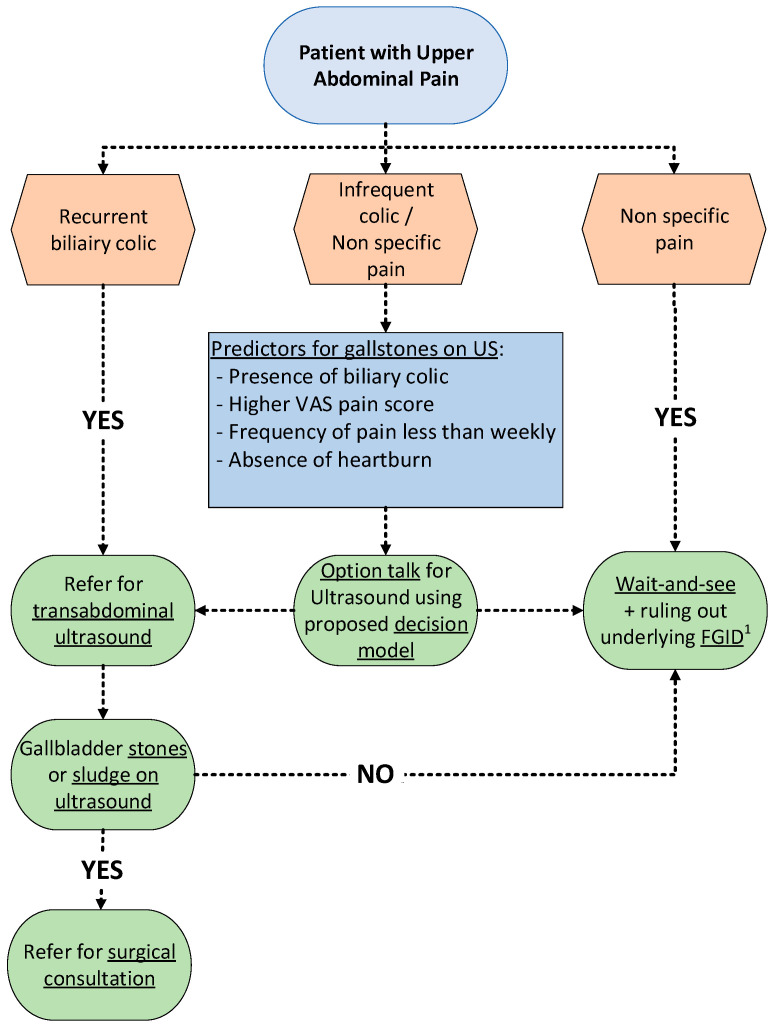
Decision tree for the diagnostic workup of patients with upper abdominal pain in primary care. During consultations, different diagnostic and treatment options and possible outcomes are open for discussion. ^1^ FGID: functional gastrointestinal diseases (i.e., functional dyspepsia and/or irritable bowel syndrome).

The diagnostic workup in the large group of patients with clinical suspicion of gallstone disease is challenging. The present study illustrates that gallstones were not confirmed in 64% of patients of with a clinical suspicion of gallstones described by the GP. The outcome of this study resulted in a model to better select patients for US. The model is based on clinical pain characteristics, frequency of pain, and the absence of heartburn. The present findings are summarized in a decision tree, which may help during the option talk with patients who report UAP on the benefit of additional diagnostics (Figure 3).

### 4.2. Strengths and Limitations

This prospective multicentre study has strengths and limitations. Our findings provided new insights into the diagnostic pathway for patients with UAP and accentuated the complexity of correctly diagnosing symptomatic cholecystolithiasis. Secondly, validated questionnaires were used to assess abdominal pain and symptoms. A limitation was the relatively small sample size of the present study [19]. However, the sample size was sufficient for the number of variables we studied, but for a more extensive study with many more predictors a larger sample will be needed [20,21,22]. Nevertheless, the AUC is good and the range is reasonable (AUC = 0.73, range 0.68–0.76). It is also considerably better at predicting the presence of gallstones than currently used ROME criteria (AUC = 0.59, range 0.57–0.61).

### 4.3. Comparison with Existing Literature

This study echoes the results of Berger et al. published in 2004. Based on their research on 233 primary care patients, the authors stated that neither biliary pain nor any other gastrointestinal symptomatology was related to gallstones consistently [23,24]. The authors concluded that before the diagnosis of symptomatic gallstones is made, other reasons for upper abdominal pain and symptoms (i.e., FGID) should be ruled out. Likewise, the present analysis revealed that the ROME III criteria alone are inadequate for predicting the presence of gallstones on US, as heartburn and the frequency of pain appeared to be important predictors [8]. Berger and colleagues already called for additional data from a randomized controlled trial to clarify the indication for cholecystectomy. Almost twenty years later, outcomes of such studies are available [10]. 

Two studies illustrated the challenge of defining symptomatic gallstone disease, and subsequently showed the negative impact of the presence of FGID on the outcome of a cholecystectomy. In 2011, Thistle et al. presented the results of their prospective study in 1008 patients undergoing cholecystectomy for symptomatic gallstone disease. Fifty-nine percent of patients were pain-free after one year of follow up. Independent predictors of pain relief were the frequency of pain less than once a month, the onset of pain less than 1 year before surgery, and nocturnal pain [25]. Moreover, functional symptoms were negatively associated with pain relief. The Dutch PERFECT trial found similar results in a multicentre cohort study of 401 gallstone patients. The baseline characteristics showed that one third of patients with gallstones also fulfil criteria for FGID. Patients with these concomitant conditions were 2.5 times less likely to become pain-free after surgery [10].

The diagnostic workup of patients with UAP generally consists of laboratory tests, abdominal US or an upper gastrointestinal endoscopy, and FGID; irritable bowel syndrome, gallstones, and dyspepsia are the most common diagnosis [26]. Speets et al. reported a diagnostic yield for the detection of gallstones on US of 8%. This study was conducted in an academic hospital with a diverse patient population [13]. The detection of gallstones on US is often coincidental, while the risk of missing the diagnosis cholecystolithiasis is relatively harmless. Less than 5% of symptomatic gallstone patients develop a biliary complication [5]. Likewise, our study shows a diagnostic yield of US (36.2%). Moreover, previous studies showed a mediocre therapeutic yield, while 40% of patients were not free of UAP after cholecystectomy. Considering these outcomes, 100 referrals for an ultrasound of patients with a suspicion of gallstone disease resulted in 14 patients being pain-free after treatment and the prevention of 1–2 biliary complications [5,9]. Therefore, informing the patient on the various causes of UAP and the different options for clinical workup including lifestyle measures is a corner stone for the treatment of patients with UAP. This form of stepped care is safe and will postpone referral for US and may prevent unnecessary surgery.

To reduce unnecessary surgery an online decision tool was recently developed to aid surgeons in selecting patients for cholecystectomy. Based on data from 1561 patients with symptomatic gallstone disease, this tool predicts the probability of pain reduction after cholecystectomy. The tool aids surgeons in deciding whether patients with uncomplicated gallstone disease will benefit from surgery. Variables included in the model illustrate that heartburn, pain scores, surgical history, and patient characteristics are valuable in predicting clinically relevant pain reduction after cholecystectomy [17].

### 4.4. Implications for Practice

The model based on this study may be helpful to move forward and facilitate an evidence-based option talk between GPs and patients with suspected gallstone disease. A proposal for a patient trajectory, including option talks for patients with UAP, is shown in Figure 3. During consultations, different diagnostic and treatment options and possible outcomes are open for discussion. The model proposed in this study and the clinical decision tool help to better select patients with symptomatic gallstone disease for cholecystectomy, and might optimise patients’ expectations and reduce ineffective use of healthcare resources [17]. The latter may be accomplished by reducing the number of USs performed in patients with UAP, for whom a laparoscopic cholecystectomy will be a mediocre solution to reduce pain. Tailored expectations regarding the outcome of the treatment helps patients to not always have to choose an operation.

## 5. Conclusions

In conclusion, this study shows that for two thirds of patients referred for US because of a suspicion of gallstones the diagnosis of symptomatic gallstone disease cannot be confirmed. The high suspicion of gallstones in patients with abdominal pain and symptoms underlines the complexity of correctly diagnosing gallstone disease in primary care. The present prediction model aids GPs in decision making, and we advise GPs to address uncertain outcomes of referral and rule out possible gastrointestinal disorders before the patient is referred for US on the suspicion of gallstones.

## Figures and Tables

**Table 1:** Baseline characteristics of patients included with and without gallstones on US. Clinical suspicion of gallstones was scored when GP referred patient for a direct-access US with the question of possible gallstone disease or sludge.

	Total (N = 177)	Gallstones (N = 64)	Absence of Gallstones (N = 113)	*p*
**Baseline characteristics**				
Age, mean (SD)	54.8 (15.4)	56.1 (14.8)	53.8 (16.0)	0.384
Sex, Female n (%)	119 (67.2)	46 (71.9)	73 (64.6)	0.322
BMI, mean (SD)	26.8 (4.7)	27.2 (4.3)	26.5 (4.9)	0.354
Consumes alcohol, n (%)	126 (71.2)	44 (68.8)	82 (72.6)	0.590
Current smoker, n (%) ^^^	28 (15.8)	4 (6.3)	24 (21.2)	**0.009**
Abdominal surgery in history, n (%)	51 (28.8)	18 (28.1)	33 (29.2)	0.879
**Abdominal pain**				
Izbicki Pain Score, median (IQR)	32.5 (26.9–40.0)	31.2 (26.8–37.6)	33.7 (26.8–42.0)	0.185
VAS pain score, median (IQR)	7.0 (4.0–8.0)	8.0 (5.5–9.0)	6.0 (4.0–7.5)	***p* < 0.001**
Biliary symptoms *				
Severe pain attacks, n (%)	128 (72.3)	50 (78.1)	78 (69.0)	0.194
Pain in right upper quadrant/epigastric region, n (%)	148 (83.6)	55 (85.9)	93 (82.3)	0.530
Duration of pain longer than 15–30 min, n (%) *	137 (77.4)	56 (87.5)	81 (71.7)	**0.020**
Biliary colic, n (%) *	90 (50.8)	40 (62.5)	50 (44.2)	**0.023**
Pain radiating to the back, n (%)	96 (54.2)	36 (56.3)	60 (53.1)	0.686
Use of pain medication, n (%)	74 (41.8)	36 (56.3)	38 (33.6)	**0.003**
Urge to move, n (%) ^β^	45 (25.4)	15 (23.4)	30 (26.5)	0.998
Weekly pain, n (%) ^β^	76 (42.9)	14 (21.9)	62 (54.9)	***p* < 0.001**
**Functional symptoms ^β^**				
Difficult defecation, n (%) ^#^	14 (7.9)	1 (1.6)	13 (11.5)	**0.026**
Diarrhoea, n (%) ^β^	12 (6.8)	3 (4.7)	9 (8.0)	0.495
Heartburn, n (%) ^β^	17 (9.6)	3 (4.7)	14 (12.4)	0.133
Abdominal bloating, n (%) ^β^	30 (16.9)	5 (7.8)	25 (22.1)	**0.026**
Nausea/Vomitus, n (%)	15 (8.5)	6 (9.4)	9 (8.0)	0.604

^^^ 1 missing; * 1 missing; ^β^ 19 Missing; ^#^ 18 missing; Abbreviations: BMI, body mass index (calculated as weight in kilograms, divided by height in meters squared): IQR, interquartile range; VAS, visual analogue scale.

**Table 2:** Predictors for the presence of gallstones in patients with clinical suspicion of gallstone disease by the GP. Multivariable logistic regression analyses with backward selection were performed to assess predictors at baseline for the detection of gallstones in patients. US-confirmed gallstones yes/no served as the outcome. The threshold for variable selection was 0.15. Model development was based on 177 patients.

Predictors	Odds Ratio (95% CI)
Intercept	
**Presence of biliary colic according to ROME criteria ^&^**	
No	1 [Reference]
Yes	1.36 (0.63–2.92)
VAS pain score squared *	
No pain	1 [Reference]
Per step	1.05 (0.99–1.11)
**Heartburn**	
No	1 [Reference]
Yes	0.61 (0.12–3.08)
**Frequency of pain is weekly**	
No	1 [Reference]
Yes	0.35 (0.15–0.83)
**AUC**	0.73 (0.68–0.76)

VAS: visual analogue scale. AUC: area under the curve. * VAS pain score was entered as squared function because of nonlinearity. ^&^ Biliary colic according to ROME criteria: severe steady pain, lasting 30 min or more, located in the epigastrium and/or right upper quadrant.

## Data Availability

Additional unpublished data related to this study are not available. Data are available on reasonable request.

## Abbreviations

US (ultrasound), UAP (Upper abdominal pain), GP (General practitioner), FGID (functional gastrointestinal diseases), IPS (Izbicki Pain score), VAS (Visual analgesic score).
